# Clinical significance of SUVmax in ^18^F-FDG PET/CT scan for detecting nodal metastases in patients with oral squamous cell carcinoma

**DOI:** 10.1186/s40064-015-1521-6

**Published:** 2015-11-24

**Authors:** Kazuhiro Kitajima, Yuko Suenaga, Tsutomu Minamikawa, Takahide Komori, Naoki Otsuki, Ken-ichi Nibu, Ryohei Sasaki, Tomoo Itoh, Kazuro Sugimura

**Affiliations:** Department of Radiology, Kobe University Graduate School of Medicine, 7-5-2 Kusunoki-cho, Chuo-ku, Kobe, 650-0017 Japan; Department of Oral and Maxillofacial Surgery, Kobe University Graduate School of Medicine, 7-5-2 Kusunoki-cho, Chuo-ku, Kobe, 650-0017 Japan; Department of Otolaryngology-Head and Neck Surgery, Kobe University Graduate School of Medicine, 7-5-2 Kusunoki-cho, Chuo-ku, Kobe, 650-0017 Japan; Department of Radiology, Division of Radiation Oncology, Kobe University Graduate School of Medicine, 7-5-2 Kusunoki-cho, Chuo-ku, Kobe, 650-0017 Japan; Department of Diagnostic Pathology, Kobe University Hospital, 7-5-2 Kusunoki-cho, Chuo-ku, Kobe, 650-0017 Japan

**Keywords:** PET/CT (positron emission tomography/computed tomography), FDG (fluorine-18-labeled fluorodeoxyglucose), SUV (standardized uptake value), Lymph node metastasis, OSCC (oral squamous cell carcinoma)

## Abstract

To retrospectively investigate the diagnostic accuracy of FDG-PET/CT relative to CT for detection of cervical node metastases in patients with oral squamous cell carcinoma (OSCC), using histologic evaluation of dissected cervical nodes as the reference standard. Thirty-six patients with OSCC who underwent neck dissection (4 bilateral, 32 unilateral; 250 nodal levels) after FDG-PET/CT. Two observers consensually determined the lesion size and SUVmax of visible cervical nodes and compared the results with pathologic findings at the nodal level. Histopathology revealed nodal metastases in 13 (36.1 %) of 36 patients and 28 (11.2 %) of 250 nodal levels. Using a best discriminative SUVmax cut-off of 3.5 for the node, the sensitivity, specificity and accuracy of FDG-PET/CT for identification of nodal metastases on a level-by-level basis were 67.9, 94.6, and 91.6 %, respectively. The corresponding figures for CT were 42.9, 96.8, and 90.8 %, respectively. The sensitivity of FDG-PET/CT was significantly better than CT (p = 0.023). Moreover, using the level-based modified SUVmax cut-off, the respective figures for FDG-PET/CT were 71.4, 95.9, and 93.2 %, with significantly higher sensitivity (p = 0.013) and accuracy (p = 0.041) than CT. FDG PET/CT with SUVmax is a useful modality for preoperative evaluation of cervical neck lymph node metastases in patients with OSCC.

## Background

Pretreatment assessment of cervical lymph node metastasis is important for therapeutic planning and prognostication in patients with oral squamous cell carcinoma (OSCC) (Snow et al. 1992). Preoperative nodal status is usually evaluated by means of clinical examinations such as palpation, computed tomography (CT), ultrasonography (US), and magnetic resonance imaging (MRI). Unfortunately, CT and MRI, which evaluate morphologic parameters such as nodal size, internal architecture and contrast enhancement pattern, have been shown to have only limited value for this purpose (Castelijins and van den Brekel 2002). At present, neck dissection with histologic examination of lymph nodes is still the most reliable staging procedure. However, it is unavoidably invasive, and therefore a noninvasive procedure capable of providing high-quality prognostic data approaching this gold standard would be of immense value.

Positron emission tomography (PET) using the glucose analog, fluorine-18-labeled fluorodeoxyglucose (FDG), is a functional imaging modality that provides information about tissue glucose metabolism. Integrated PET/CT has been applied successfully for evaluation of squamous cell carcinoma of the head and neck (HNSCC), and recent reports have suggested that it is also useful for evaluation of nodal involvement in OSCC. There is growing evidence that FDG-PET or PET/CT is a more reliable and accurate imaging tool than CT for evaluation of cervical neck lymph node metastasis in OSCC (Matsubara et al. 2012; Ng et al. 2005; Yamazaki et al. 2008). On the other hand, several reports have indicated that FDG-PET or PET/CT offers no advantage, especially for evaluation of the N0 neck in early OSCC (Krabbe et al. 2008; Nahmias et al. 2007; Schöder et al. 2006), and therefore its diagnostic value remains controversial.

The maximum standardized uptake value (SUVmax) is widely used for measuring the uptake of FDG by malignant tissue (Gambhir 2002). Increased FDG uptake values reflect the viability of cancer cells, and can be imaged and quantified using PET. Recent studies of OSCC have demonstrated that the SUVmax of the primary tumor is related to proliferative cell activity and cellularity, and also to the prognosis of patients (Suzuki et al. 2009). However, few studies have reported the clinical significance of SUVmax for diagnosis of cervical lymph node metastasis in OSCC (Matsubara et al. 2012), and thus the true usefulness of SUVmax in this context remains unclear.

Clinically, it is recognized that level IIa nodes located around the internal vein and anterior spinal accessory nerve at levels between the skull base and the hyoid bone often show relatively higher nonspecific FDG uptake due to reactive hyperplasia in comparison with nodes at other levels. Therefore, as reported previously by Jeong et al. in a study of patients with HNSCC, it may be better to use a SUV cut-off that differs according to the node level, for differentiating malignant from benign lymph nodes (Jeong et al. 2007).

The aim of the present study was to investigate the diagnostic accuracy of cervical node evaluation by FDG-PET/CT using SUVmax in patients with OSCC in comparison with CT, and to examine the clinical utility of level-based modified SUVmax cut-off values.

## Methods

### Patients

This retrospective study approved by our institutional review board involved 36 patients (23 males, 13 females; average age at diagnosis 67.3 years, range 37–88 years), from whom informed consent was waived. All of the study subjects with biopsy-proven OSCC underwent resection of the primary tumor and cervical node dissection within 4 weeks after undergoing FDG-PET/CT examinations at our institution between November 2011 and July 2014. The primary tumor was located in the oral tongue in 16 patients, the gingiva in 12, and the floor of mouth in 8. Patient demographics and clinicopathologic variables are shown in Table 1.

**Table 1:** Patient characteristics

Character	Value
Sex	
Male	23
Female	13
Age	
Mean	67.3 ± 10.0
Range	37–88
Primary tumor sites	
Oral tongue	16
Gum	12
Floor of mouth	8
T classification	
T1	5
T2	16
T3	7
T3	8
N classification	
N0	23
N1	3
N2a	1
N2b	7
N2c	2
Neck dissections	
Ulilateral	32
Bilateral	4
Type of neck dissection	
SOHND (levels I–III)	15
Extended SOHND (levels I–IV)	5
MRND, typeIII(levels I–V)	19
LND (levels II–IV)	1

*SOHND* supramohyoid neck dissection, *MRND* modified radical neck dissection with preservation of sternocleidomastoid muscle, internal jugular vein, and spinal accessory nerve, *LND* lateral neck dissection

The studied patients underwent unilateral (n = 32) or bilateral (n = 4) neck dissection. A total of 40 sites in the neck lymph node basin were subjected to type III modified radical dissection (n = 19), supraomohyoid neck dissection (n = 15), extended supraomohyoid neck dissection (n = 5), or lateral neck dissection (n = 1). Pathologically, 3 patient had T1, 9 had T2, 13 had T3, and 11 had T4 tumors; nodal involvement was N0 in 12 patients, N1 in 12, N2b in 9, and N2c in 3.

### FDG-PET/CT

Whole-body imaging was performed using a combined PET/CT scanner (Discovery PET/CT 690, GE Healthcare, Waukesha, WI, USA). CT covered a region ranging from the meatus of the ear to the midthigh. The technical parameters of the 16-detector-row helical CT scanner were a helical pitch of 28 or a beam pitch of 1.75, a gantry rotation speed of 0.6 s, and a slice thickness of 3.27 mm. The PET component of the combined imaging system allowed simultaneous acquisition of 47 transaxial PET images with an interslice spacing of 3.27 mm in one bed position, and provided an image from the meatus of the ear to the midthigh with 7–8 bed positions. The transaxial field of view and axial field of view of the PET images reconstructed for fusion were 60 and 15.0 cm, respectively, with a matrix size of 192 × 192. To avoid artifacts caused by the urinary tract, patients were asked to drink 500 ml of water 1–2 h prior to image acquisition, and to void just before the start of acquisition. After at least 4 h of fasting, patients received an intravenous injection of 222–333 MBq (6–9 mCi) of ^18^F-FDG. The blood glucose levels were checked in all patients before FDG injection, and no patients showed a blood glucose level of more than 200 mg/dL.

About 50 min later, initial low-dose non-enhanced CT was performed at 120 kV and Smart mA (20–120 mA, Noise Index 30) using the normal expiration position for attenuation correction of the PET image. A whole-body emission PET scan was performed immediately after the low-dose non-enhanced CT scan, with a 2-min acquisition per bed position using the three-dimensional acquisition mode. Attenuation-corrected PET images were reconstructed with an ordered-subset expectation maximization iterative reconstruction algorithm, VUE Point FX-S, with TOF and sharp IR (18 subsets, 2 iterations).

For image fusion, a 3.27-mm slice was reconstructed. The CT and PET images were transferred to a commercially available workstation (Advantage Windows Workstation, version 4.5, GE Healthcare Technology) in order to access all of the data.

### Image analysis

PET images were interpreted retrospectively by two experienced nuclear medicine physicians. For semiquantitative analysis of FDG uptake, regions of interest (ROIs) were defined on the target lesions (primary lesion and neck lymph node) in the transaxial PET images. The maximum standardized uptake value (SUV) was calculated for quantitative analysis of tumor FDG uptake, as follows:

$${\text{SUV}} = {\text{C }}\left( {{\text{kBq}}/{\text{ml}}} \right)/{\text{ID }}\left( {\text{kBq}} \right)/{\text{body weight }}\left( {\text{kg}} \right)$$

where C is the tissue activity concentration measured by PET, and ID is the injected dose.

For nodal disease, the highest SUVmax was used for quantitative evaluation.

Nodes were considered to harbor metastasis if their longest axial diameter was >15 mm for levels I and II or >10 mm for levels III-V, if they appeared spherical (rather than flat or bean-like) in shape, or showed rim enhancement with central necrosis or cystic degeneration, and if they were abnormally grouped (Sakai et al. 2000; Som et al. 2000).

If there were multiple lymph nodes at a specific level, the node suspected to have the highest malignant potential on CT or showing the highest SUVmax on PET/CT was assessed.

### Surgical procedure and histology

Neck dissection was planned by our head and neck surgical team based on the clinical and imaging findings (Ferlito et al. 2006). Supraomohyoid neck dissection (SOHND, levels I–III) was performed for patients who were node-negative in the neck, or who had a single positive node in the upper neck. Extended supraomohyoid neck dissection (extended SOHND, levels I–IV) or modified radical neck dissection (MRND, levels I–V) was performed for patients with >1 involved node or extracapsular nodal spread, depending on the extent of the cervical adenopathy. Bilateral neck dissection was performed for patients in whom the primary tumor crossed the midline, or those considered likely to have node metastases in the contralateral neck. The operative surgeon labeled the primary tumor and neck dissection specimens so as to allow reference to the schema used for interpretation of the FDG-PET/CT studies. Lymph nodes and tumors were dissected from the specimens and stained with hematoxylin and eosin for histologic analysis. Serial histologic sections were used. An experienced pathologist examined the specimens and recorded the number, size, and capsular penetration of the affected nodes.

### Statistical analysis

On the basis of the neck level system (Sakai et al. 2000), we compared the results of preoperative FDG-PET/CT and CT examinations with those of the corresponding histopathologic examinations. Because precise spatial correlation between PET/CT and histopathology is impossible, analysis was restricted to nodal levels and neck sides. If the findings of PET were suggestive of metastasis, and if histopathology showed at least one lymph node with metastasis at a given nodal level in the neck, a true positive finding was recorded, regardless of the number of metastatic foci at that level.

Receiver operating characteristic (ROC) curve analysis was performed to evaluate whether SUVmax was able to allow diagnosis of nodal metastasis and to identify the best cutoff value.

Analysis was performed on a patient, neck side, and lymph node level basis. Sensitivity, specificity, positive predictive value (PPV), negative predictive value (NPV), and accuracy were calculated using standard statistical formulae, and the 95 % confidence interval (CI) was determined for each parameter. Differences at p < 0.05 were considered statistically significant. All analyses were performed using the SAS software package version 9.2 (SAS Institute, Cary, NC, USA).

## Results

Overall, 40 neck sides were dissected (32 unilateral, 4 bilateral), involving 250 nodal levels and a total of 1257 lymph nodes (mean, 31.3 lymph nodes per neck side). Histopathologic analysis revealed lymph node metastases in 13 of the 36 patients (36.1 %), 15 of the 40 neck sides (37.5 %), and 28 of the 250 nodal levels (11.2 %). Overall, metastases were found in 35 of the 1257 dissected lymph nodes (2.8 %).

Cervical lymph nodes were dissected at 250 neck levels (Ia:Ib:IIa:IIb:III:IV:V = 37:42:40:39:40:27:25), and malignant cells were found at 28 neck levels (Ia:Ib:IIa:IIb:III:IV:V = 2:9:11:1:5:0:0).

### Primary tumors

All 36 primary tumors were clearly identified by FDG-PET; the intensity of FDG uptake in these lesions ranged from moderate to very intense, with an SUVmax range of 6.41–28.73 (mean, 15.5 ± 6.6).

### Metastatic neck disease

#### Level-by-level analysis

The SUVmax of malignant lymph nodes showing significant FDG uptake (mean SUVmax, 6.03 ± 4.22; range 1.13–17.02) was significantly higher than that of benign lymph nodes (mean SUVmax, 1.98 ± 0.84: range 0.79–5.24; p < 0.0001). Nodes with a SUVmax of >5.25 were all confirmed pathologically to harbor metastases. The SUVmax of metastatic lymph nodes with and without extracapsular spread were 11.41 ± 3.93 (8.09–17.02) and 5.13 ± 3.60 (1.13–12.17), respectively (p = 0.040).

**Fig. 1:**
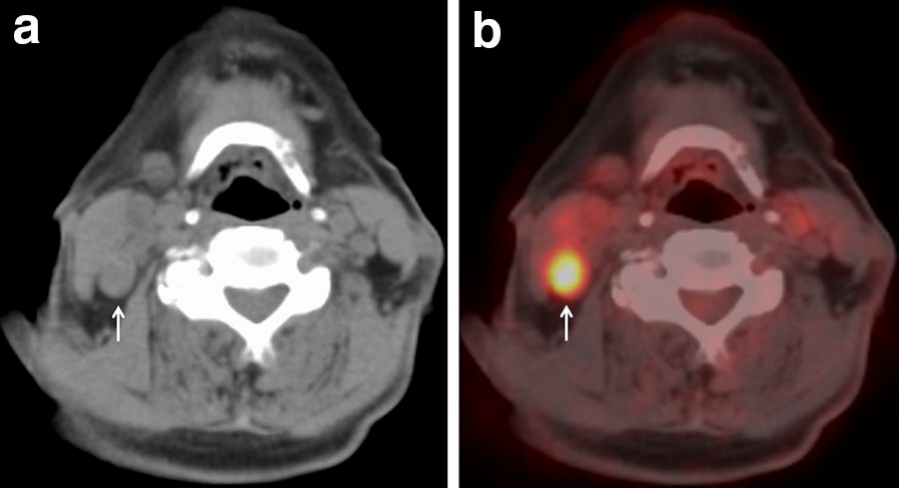
A 77-year-old man with level IIa node metastasis arising from cancer of the tongue (pT4N2b). **a** CT of PET/CT shows one swollen lymph node 20 mm in longest diameter at right level IIa (*arrow*), suggesting the presence of nodal cancer spread. **b** FDG-PET/CT shows intense FDG uptake (SUVmax:11.91) corresponding to the right level IIa node seen in **a** (*arrow*), suggesting the presence of nodal cancer spread. Examination of the histopathological specimen confirmed extensive lymph node involvement by cancer in this node. Both CT and FDG-PET/CT were true-positive

**Fig. 2:**
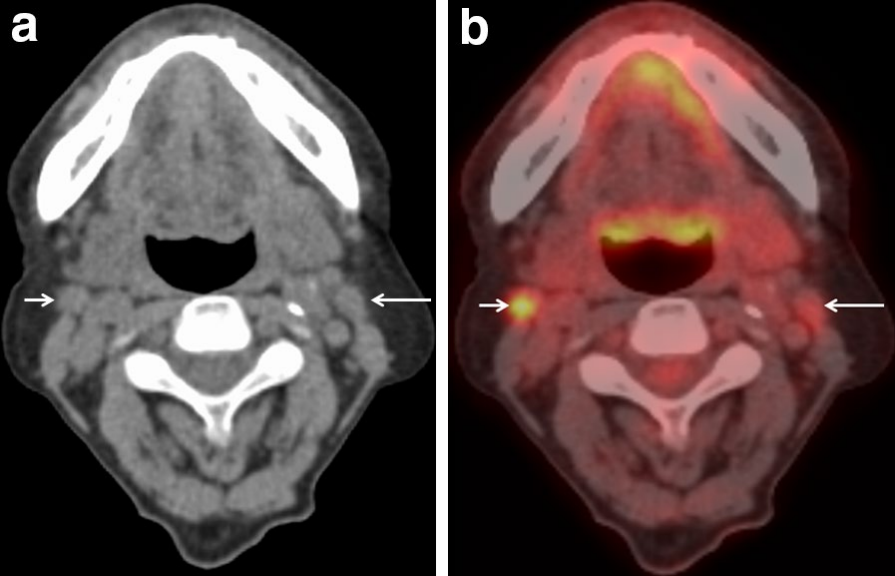
A 63-year-old man with right level IIa node metastasis arising from cancer of the gum (pT3N2c). **a** CT of PET/CT shows one swollen lymph node 10 mm in longest diameter at right level IIa (*short arrow*) and one swollen lymph node 11 mm in longest diameter at left level IIa (*long arrow*), suggesting absence of nodal cancer spread. **b** FDG-PET/CT shows moderate FDG uptake (SUVmax:5.34) corresponding to the right level IIa node seen in a (*short arrow*), suggesting the presence of nodal cancer spread. And mild FDG uptake (SUVmax:2.31) corresponding to the left level IIa node seen in **b** (*long arrow*), suggesting absence of nodal cancer spread. Histopathological examination of the specimen confirmed extensive lymph node involvement by cancer in only the right node. CT gave a false-negative result for the right node, whereas FDG-PET/CT gave a true-positive result. Both CT and FDG-PET/CT gave true-negative results for the left node

**Fig. 3:**
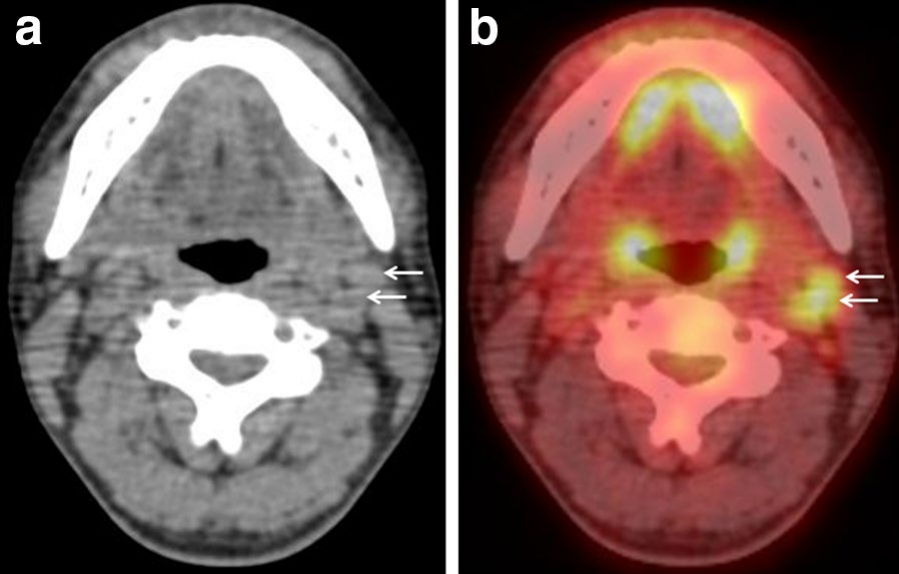
A 66-year-old man without neck node metastasis arising from the tongue (pT2N0). **a** CT of PET/CT shows two swollen lymph nodes 10 and 9 mm in longest diameter at left level IIa (*arrows*), suggesting the absence of nodal cancer spread. **b** FDG-PET/CT shows moderate FDG uptake (SUVmax:3.71 and 3.56) corresponding to the two left level IIa nodes seen in a (*arrows*). Examination of the histopathological specimen confirmed no lymph node metastasis. CT gave true-negative result. FDG-PET/CT using SUVmax cut-off (3.5) gave false-positive result, whereas FDG-PET/CT using modified SUVmax cut-off (4.0) gave true-negative result

Using a best discriminative SUVmax cut-off of 3.5 for discriminating metastatic from benign cervical nodes based on ROC curve analysis with an area under ROC curve (AUC) of 0.874, the sensitivity, specificity and accuracy of FDG-PET/CT for identification of nodal metastases on a level-by-level basis were 67.9 % (19/28), 94.6 % (210/222), and 91.6 % (229/250), respectively. The corresponding figures for CT were 42.9 % (12/28), 96.8 % (215/222), and 90.8 % (227/250), respectively. The sensitivity of FDG-PET/CT was significantly better than that of CT (p = 0.023), but the differences in specificity and accuracy were not statistically significant (Table 2). Three representative cases are shown in Figs. 1, 2 and 3: Fig. 1 shows that both CT and FDG-PET gave true-positive results, whereas Fig. 2 shows that CT gave a false-negative result and FDG-PET a true-positive result.

**Table 2:** Level-by-level diagnostic performance of three methods (CT, PET/CT with best SUVmax cut-off, and PET/CT with level-based modified SUVmax cut-off)

	TP	FN	TN	FP	Sensitivity	Specificity	PPV	NPV	Accuracy
					95 % CI	95 % CI	95 % CI	95 % CI	95 % CI
CT	12	16	215	7	42.9^a,b^	96.8	63.2	93.1	90.8^c^
					24.6–61.2	94.5–99.1	41.5–84.9	89.8–96.4	87.2–94.4
FDG-PET/CT using best SUVmax cut-off (3.5)	19	9	210	12	67.9^a^	94.6	61.3	95.9	91.6
					50.6–85.2	91.6–97.6	59.8–62.8	93.3–98.5	88.2–95.0
FDG-PET/CT usinglevel-based modified SUVmax cut-off	20	8	213	9	71.4^b^	95.9	69	96.4	93.2^c^
					54.7–88.1	93.3–98.5	67.6–70.5	93.9–98.9	90.1–96.3

*TP* true positive, *FN* false negative, *TN* true negative, *FP* false positive, *PPV* positive predictive value, *NPV* negative predictive value, *CI* confidence interval

^a^The sensitivity of FDG-PET/CT with best SUVmax cut-off (3.5) was significantly higher than that of CT (p = 0.023)

^b^The sensitivity of FDG-PET/CT with level-based modified SUVmax cut-off was significantly higher than that of CT (p = 0.013)

^c^The accuracy of FDG-PET/CT with level-based modified SUVmax cut-off was significantly higher than that of CT (p = 0.041)

#### Level-based analysis

Table 3 shows the level-based SUVmax for metastatic and benign lymph nodes, and the level-based best SUVmax cut-off for discriminating metastatic from benign nodes. Because the SUVmax for benign nodes at level IIa was slightly high (mean: 2.85, range 0.95–5.24), the optimum SUVmax cut-off was slightly high (4.0). Because the SUVmax for metastatic nodes at level IIb was relatively low (2.84), the optimum SUVmax cut-off was slightly low (2.8). Table 3 also shows the diagnostic performance of FDG-PET/CT using the level-based modified SUVmax cut-off for identification of nodal metastases in comparison with the figures for CT. Using the level-based modified SUVmax cut-off (3.5 for levels Ia, Ib, III, IV, and V, 4.0 for level IIa, and 2.8 for level IIb), the sensitivity, specificity, and accuracy of FDG-PET/CT were 71.4 % (20/28), 95.9 % (213/222), and 93.2 % (233/250), respectively, the sensitivity (p = 0.013) and accuracy (p = 0.041) being significantly higher than those for CT (Table 2). Figure 3 shows that FDG-PET using SUVmax cut-off (3.5) gave a false-positive result and only both CT and FDG-PET using modified SUVmax cut-off (4.0) gave a true-negative result.

**Table 3:** Level-based analysis

	SUVmax		Best Cut-off SUVmax	Sensitivity	Specificity	Accuracy	Modality
	Mean	Range					
Level Ia (n = 37)							
Metastatic nodes (n = 2)	3.14 ± 1.80	1.86–4.41	3.5	50 % (1/2)	100 % (35/35)	97.3 % (36/37)	PET
Benign nodes (n = 35)	1.71 ± 0.66	1.0–3.32		0 % (0/2)	100 % (35/35)	94.6 % (35/37)	CT
Level Ib (n = 42)							
Metastatic nodes (n = 9)	4.83 ± 3.90	1.13–10.27	3.5	66.7 % (6/9)	93.9 % (31/33)	88.1 % (37/42)	PET
Benign nodes (n = 33)	2.12 ± 0.89	0.98–4.4		22.2 % (2/9)	93.9 % (31/33)	78.6 % (33/42)	CT
Level IIa (n = 40)							
Metastatic nodes (n = 11)	7.77 ± 4.51	3.51–17.02	4.0	72.7 % (8/11)	90.0 % (26/29)	85.0 % (34/40)	PET
Benign nodes (n = 29)	2.85 ± 1.06	0.95–5.24		63.6 % (7/11)	90.0 % (26/29)	82.5 % (33/40)	CT
Level IIb (n = 39)							
Metastatic nodes (n = 1)	2.84	2.84	2.8	100 % (1/1)	92.1 % (35/38)	92.3 % (36/39)	PET
Benign nodes (n = 38)	2.85 ± 1.06	0.95–5.24		100 % (1/1)	94.7 % (36/38)	94.8 % (37/39)	CT
Level III (n = 40)							
Metastatic nodes (n = 5)	6.13 ± 4.44	1.53–11.91	3.5	80.0 % (4/5)	97.1 % (34/35)	95.0 % (38/40)	PET
Benign nodes (n = 35)	1.76 ± 0.84	0.79–3.77		40.0 % (2/5)	100 % (35/35)	92.5 % (37/40)	CT
Level IV (n = 27)							
Metastatic nodes (n = 0)				–	100 % (27/27)	100 % (27/27)	PET
Benign nodes (n = 27)	1.69 ± 0.45	0.95–2.67		–	100 % (27/27)	100 % (27/27)	CT
Level V (n = 25)							
Metastatic nodes (n = 0)					100 % (25/25)	100 % (25/25)	PET
Benign nodes (n = 25)	14.44 ± 0.42	0.93–2.52			100 % (25/25)	100 % (25/25)	CT

#### Patient-based analysis

The sensitivity, specificity and accuracy of FDG-PET/CT for identification of nodal metastases on a patient-by-patient basis were 84.6 % (11/13), 87.0 % (20/23), and 86.1 % (31/36), respectively, and the corresponding figures for CT were 76.9 % (10/13), 91.3 % (21/23), and 86.1 % (31/36), respectively. Sensitivity and specificity for the two methods were almost the same and accuracy was exactly the same.

## Discussion

In the present study, we compared the diagnostic performance of SUVmax between FDG-PET and CT for evaluation of cervical lymph node metastases in OSCC using histopathology as the gold standard. Although the specificity of FDG-PET on level-by-level basis was slightly inferior to that of CT (94.6 vs. 96.8 %), the sensitivity of SUVmax was significantly higher than that of CT (67.9 vs. 42.9 %, p = 0.023). Moreover, FDG-PET/CT with level-based modified SUVmax cut-off values had significantly higher sensitivity (71.4 vs. 42.9 %, p = 0.013) and accuracy (93.2 vs. 90.8 %, p = 0.041) than CT. Whereas, there was no difference in accuracy on patient-based analysis.

Currently, CT and MRI are commonly used for evaluation of the primary tumor and cervical node status. These modalities characterize the cervical lymph nodes on the basis of morphological criteria such as node size, the presence of central necrosis, and the presence of indistinct nodal margins. The reported sensitivity and specificity of CT and MRI for detection of cervical lymph node metastases in OSCC are 36–78 and 47–99 %, respectively (Castelijins and van den Brekel 2002; Conti et al. 1996; Hannah et al. 2002; Krabbe et al. 2008; Matsubara et al. 2012; Ng et al. 2005; Yamazaki et al. 2008). Doppler US with fine-needle aspiration can overcome some of these limitations, but the results are dependent on the skill level of the sonographer, and this may be impractical in some cases because the number of questionable nodes may be high.

Several studies have evaluated the diagnostic performance of FDG-PET for detecting cervical lymph node metastases of OSCC (Krabbe et al. 2008; Matsubara et al. 2012; Nabmias et al. 2007; Ng et al. 2005; Yamazaki et al. 2008; Schöder et al. 2006). Data from those studies demonstrated large variations in sensitivity and specificity, being 50–95 % and 82–99 %, respectively. Sun et al. (Sun et al. 2015) reviewed 24 studies of 1270 patients with HNSCC to assess nodal metastasis and reported that the mean (95 % CI) pooled per-patient, per-neck-side, and per-neck-level sensitivities/specificities of FDG-PET/CT were 91 % (82–95 %)/87 % (80–92 %), 84 % (75–90 %)/83 % (77–88 %), and 80 % (71–87 %)/96 % (94–97 %), respectively. Across 13 studies (3,460 neck levels) with per-neck-level data, the sensitivity and specificity of FDG-PET/CT were 84 % (72–91 %) and 96 % (95–97 %), and of conventional imaging (CT, MRI, and CT/MRI) were 63 % (53–72 %) and 96 % (95–97 %), respectively.

Only one study by Matsubara et al. (2012) has used SUVmax for evaluation of cervical lymph nodes of OSCC, and the authors proposed the new assessment method combining SUV and nodal size. Jeong et al. (2007) have used level-based SUV for evaluation of cervical lymph nodes in patients with HNSCC, including cancers of the oral cavity, larynx, oropharynx, hypopharynx, and unknown primary sites. Although in our present series we identified an optimum SUVmax cut-off value of 3.5 by ROC curve analysis, this figure was coincidentally the same as that reported by Jeong et al. (2007). Similarly to our study, they adopted 4.0 as the threshold SUV for jugulodigastric nodes, which is where reactive hyperplasia frequently occurs, as was seen in our series. Because the SUVmax of benign nodes at level IIa tends to be slightly high, it is better to establish a slightly higher SUVmax cut-off for this level.

Although FDG-PET is more useful than CT for diagnosis of cervical lymph node metastasis, the diagnostic capability of FDG-PET is limited not only by cellular activity but also by tumor volume. FDG uptake by small deposits of tumor cells is often poorly depicted owing to partial volume effects (Takamochi et al. 2005). Moreover, its registration is limited to a certain lymph node size, because the spatial resolution of recent PET scanners is technical limited to 4–5 mm (Yamazaki et al. 2008). A previous study has suggested that occult metastases have tumor involvement extending over only 1–2 mm (Stoeckli et al. 2002). Therefore, FDG-PET scanning may be unable to detect occult nodal metastases. In the future, the development of dual time point PET, new tumor-specific tracers and PET scanners with a higher resolution may increase the potential to detect occult lymph node metastases.

In the present series, false-positive findings were encountered in several cases. The intensity of tracer uptake by inflammatory lymph nodes is virtually the same as that in metastatic lesions. Common exposure to carcinogens in tobacco smoke and alcohol may be responsible for chronic, low-level lymphadenitis in patients with head and neck cancer. Accordingly, histologic analysis of false-positive nodes showed follicular and parafollicular hyperplasia of lymphoid tissue in all false-positive cases. Similar histologic changes in false PET-positive nodes have been reported in lung cancer staging studies (Gonzalez-Stawinski et al. 2003).

There were several limitations to the present study. First, it had a retrospective design and involved a relatively small number of patients at a single institution. Second, as every patient was a candidate for surgery including neck dissection, a patient selection bias was unavoidable. Third, full-dose contrast-enhanced CT was performed for 18 patients (50 %) at the time of FDG-PET/CT and was not used in our analysis. Given the low-dose non-enhanced CT used, visualization of nodes on CT may have been reduced compared with visualization of nodes on full-dose contrast-enhanced CT. Fourth, although node-based analysis is an ideal approach, it was very difficult to correlate any given lymph node depicted in an imaging study with the same node in a neck dissection specimen. Therefore, correlation of imaging results with pathological findings based on cervical level may be more reasonable if it is done in terms of sensitivity and specificity. Fifth, the appropriate cutoff values in any study must be determined for each individual PET scanner at each institution.

## Conclusion

FDG PET/CT with SUVmax, especially the modified SUVmax cut-off optimal for each cervical level, is a very useful tool for preoperative evaluation of cervical lymph node metastasis in patients with OSCC. However, its sensitivity was still not high enough to replace pathologic lymph node staging based on neck dissection.

This study was approved by the appropriate Ethics Committee (Ethics Committee of the Kobe University Graduate School of Medicine) and thus performed in accordance with the ethical standards laid down in the 1964 Declaration of Helsinki and its later amendments.

All persons gave their informed consent prior to inclusion in the study. Details that might disclose the identity of the subjects under study have been omitted.

Current study has been performed in accordance with the ethical standards.
